# Contaminants of Emerging Concern (CECs): Occurrence and Fate in Aquatic Ecosystems

**DOI:** 10.3390/ijerph182413401

**Published:** 2021-12-20

**Authors:** Paolo Pastorino, Antoni Ginebreda

**Affiliations:** 1 The Veterinary Medical Research Institute for Piemonte, Liguria and Valle d’Aosta, Via Bologna 148, 10154 Torino, Italy; 2 Department of Environmental Chemistry, Institute of Environmental Assessment and Water Studies (IDAEA-CSIC), Jordi Girona 18–26, 08034 Barcelona, Spain; agmqam@cid.csic.es

Contaminants of emerging concern (CECs) are typically divided into chemicals, as they are properly called, and biological CECs, such as pathogens [1]. CECs comprise a vast array of contaminants that have only recently appeared in water, or that are of recent concern because they have been detected at concentrations significantly higher than expected, and/or their risk to human and environmental health may not be fully understood [2,3]. CECs span natural and artificial chemical substances and their by-products, comprising pharmaceuticals, personal care products (PPCPs), flame retardants (FRs), pesticides, artificial sweeteners (ASWs), nanoparticles, microplastics and their transformation products, but also antibiotic resistant bacteria (ARB), antibiotic resistant genes (ARG) and, more recently, the SARS-CoV-2 virus [4,5,6,7,8,9,10,11,12,13,14,15].

There are many CECs and PPCPs that act as so-called endocrine disruptors (EDCs) [16]. EDCs are compounds that alter the normal functions of hormones, resulting in a variety of health effects. EDCs can alter hormone levels, leading to reproductive effects in aquatic organisms, and evaluating these effects may require testing methodologies not typically available, along with endpoints not previously evaluated using current guidelines [17].

Another most serious risk for freshwater and marine ecosystems, and consequently human health, derives from the occurrence of pathogens (parasites and bacteria), especially ARB and ARGs, which are now widespread throughout the aquatic environment and pose a serious emerging risk for aquatic organisms (especially fish) and human health [18].

It is important to point out that the number of CECs is continuously evolving as new chemical compounds are produced, and improvements in chemical analysis increase our understanding of the effects of current and past contaminants on human and environmental health. Moreover, as the climate and environment change, humans, plants, and animals increasingly migrate, allowing disease-causing organisms of all kinds to find new areas and new hosts to live in. This leads to the frequent emergence of new diseases [19].

Introduction routes into the aquatic environment for CECs consist of point and non-point sources [20]. Major point sources include wastewater treatment plants (WWTPs), industries and hospitals [21]. Regarding the level of persistence, some CECs pass unaltered through WWTPs, and therefore, they are released into different aquatic environments [22]. Many other CECs, though not persistent *per se*, owing to their continuous release into the environment, can be truly qualified as “pseudo-persistent”. Although CECs are ubiquitous and cause blanket exposure with chronic, subtle impacts on human and environmental health, little is known about their occurrence in both biotic and abiotic matrices; therefore, data are needed to assess the risks associated with their presence [23,24].

Biological assessment is crucial for measuring the ecological integrity of aquatic ecosystems and for protecting aquatic and human life. Aquatic organisms may transfer the contaminants that they bioaccumulate from water or sediment to the fish that forage on them, and then to the humans who consume the fish [25]. The extent to which these sediment-associated contaminants can move through the aquatic food chain, and thus potentially affect organisms at higher trophic levels, is critical for environmental decision-making [26].

CECs persist for long periods in sediments, where bottom-dwelling animals accumulate them and pass them up the food chain to fish [27]. CECs levels can increase as they move up the food chain, resulting in top predators in a food chain having levels several orders of magnitude higher than the water. CEC levels in fish tissues are also an important indicator of the health of waterbodies and the risk to human health from consuming fish [24].

It is well documented that CECs are susceptible to poor removal during the conventional WWTPs, which introduce them back into the environment at concentration levels ranging from ng/L (i.e., carbamazepine) up to mg/L (i.e., acesulfame). On this path, the limited knowledge of SARS-CoV-2’s behavior in wastewater makes the proper treatment of wastewater difficult, leaving a gap that should be taken and studied to improve the technologies proposed to eliminate the presence of the virus in aquatic compartments [28].

The European Union has long recognized the importance of environmental monitoring for recording the combined exposure of the environment and humans to contaminants and the unique role that these instruments play in identifying exposure to substances problematic for human health and the environment [29]. However, the current EU legislation relative to the water environment (Water Framework Directive and other daughter directives) is incomplete, not only because of the limited number of substances included compared to the huge amount used by our society (to date, only 45 priority substances have fixed environmental quality standards), but also because any mixture effects resulting from joint occurrence have been overlooked [30]. As the human population continues to increase, as industrial development, energy and land use continue to expand, and despite advances in pollution control, the continued production of pollution remains inevitable. Thus, the need for environmental monitoring is still as great as ever. The current body of evidence on the nature and spread of CECs is sparse, and further data are needed. Therefore, this Special Issue aims to focus on the occurrence and fate of CECs in the aquatic environment.

Contributions (i.e., original article, review, systematic review, communication, brief report, case report, discussion) may include, but are not limited to:

- occurrence of CECs in the aquatic environment;
- measurement techniques to investigate CECs;
- integration of multiple stressors with CEC exposure in aquatic systems;
- lethal and sublethal effects of CECs on aquatic organisms;
- complexity of mixtures of CECs in the aquatic environment;
- trophic consequences of CEC exposure across aquatic food webs;
- duration of CEC exposure within and across generations in aquatic species;
- pressure caused by antibiotic and other antimicrobial agents on the acquisition and spread of antibiotic resistance in the aquatic environment;
- plastics, microplastics and nanoplastics;
- CECs in wastewater;
- remediation technologies involved in the treatment and removal of pollutants;
- emerging pathogens for aquatic organisms and public health.
